# A Web-Based Platform for Quantitative Assessment of Change Detection Using Rao’s Q Index in Remote Multispectral Sensing Data

**DOI:** 10.3390/s26092665

**Published:** 2026-04-25

**Authors:** Rafaela Tiengo, Silvia Merino-De-Miguel, Jéssica Uchôa, Artur Gil

**Affiliations:** 1 Departamento de Ingeniería y Gestión Forestal y Ambiental, ETSIMFMN—Escuela Técnica Superior de Ingeniería de Montes, Forestal y del Medio Natural, Universidad Politécnica de Madrid, 28040 Madrid, Spain; r.dtiengo@alumnos.upm.es (R.T.); silvia.merino@upm.es (S.M.-D.-M.); 2 Escuela Politécnica de Cáceres, Universidad de Extremadura, Avenida de la Universidad s/n, 10003 Cáceres, Spain; jegarciau@alumnos.unex.es; 3 IVAR—Research Institute for Volcanology and Risk Assessment, University of the Azores, 9500-321 Ponta Delgada, Portugal

**Keywords:** remote sensing, web-based geospatial analysis, Rao’s Q index, land use, land cover, change detection

## Abstract

This study presents the development and implementation of a web-based geospatial platform for the quantitative assessment of land use and land cover change (LULCC) based on multispectral satellite images. The system operationalizes the Rao spectral diversity metric (Rao’s Q) to detect and quantify LULCC resulting from different environmental agents. The platform supports single-band (classic mode) or multi-band (multidimensional mode) processing. Its main functionalities include the interactive de-limitation of areas of interest (AOI) and calendar-based temporal selection, allowing analyses to be performed at discrete time points or at defined intervals. Among the tools available in the application are the automated calculation of Rao’s Q surfaces and maps of change between pairs of dates. Additionally, the platform allows the selection of several spectral indices, with the aim of supporting ecosystem monitoring and the characterization of the Earth’s surface. In the use case demonstration (Reykjanes Peninsula volcanic eruption of February 2024), the Rao’s Q method applied to Sentinel-2 SWIR imagery demonstrated strong performance in lava flow detection, with the multidimensional approach (bands 11 + 12) achieving the most balanced results (OA = 83.0%, PA = 84.0%, UA = 82.4%), while band 11 alone yielded the highest precision (UA = 97.4%). By integrating spatiotemporal analysis, spectral diversity metrics, and spectral indices into an accessible and extensible framework, the platform constitutes a robust tool for monitoring LULCC and assessing environmental impacts.

## 1. Introduction

The growing availability of geospatial technologies and remote sensing (RS) data has significantly enhanced the capacity to analyze and understand spatial phenomena across diverse fields [1]. Over the past decades, advances in satellite imaging, data processing, and digital mapping have expanded the role of geographic information in risk assessment and natural hazards monitoring [2,3,4,5,6,7,8], agricultural management [9,10], urban planning [11,12,13], and natural resource assessment [14,15,16]. As global challenges increasingly demand timely and spatially explicit information, the integration of satellite-derived datasets with modern information systems has become essential [16,17]. In this context, the broader landscape of geospatial innovation provides a foundation upon which contemporary analytical tools such as Web-Based Geographic Information Systems (WebGIS), continue to evolve and gain relevance [18,19,20,21].

In recent years, the processing of RS data has largely depended on traditional desktop environments with limited computational capacity. Such reliance has imposed notable limitations in handling extensive datasets, storage demands, and complex analytical tasks. However, these constraints have been progressively mitigated by advancements in internet technologies and cloud-computing infrastructures.

The integration of internet technologies with cloud-computing infrastructures is profoundly transforming contemporary scientific research. Through geospatial cloud platforms such as Google Earth Engine (GEE), researchers can efficiently and inexpensively access extensive collections of geographic data covering numerous thematic domains. In addition, the computational capabilities provided by cloud-based environments (e.g., Google Colab) enable the execution of complex analyses and the production of high-resolution spatial outputs [22].

In addition to this, WebGIS has emerged as a key technological development in the dissemination and analysis of geospatial information [17,19,21,23]. Defined as Geographic Information System (GIS) platforms accessible through web interfaces, WebGIS environments enable users to visualize, query, and interpret spatial data without the need for specialized desktop software or advanced computational resources [17,24]. Their architecture facilitates real-time data access, multiuser interaction, and seamless integration with distributed databases and web services, thereby enhancing the efficiency and scalability of geospatial workflows [8,25,26]. As a result, WebGIS has become an essential instrument for supporting decision-making processes in both scientific and operational contexts, offering greater accessibility, flexibility, and reach compared with traditional GIS solutions [18,19].

Despite the growing availability of satellite imagery and geospatial datasets, significant barriers persist in translating these resources into actionable information for a broad range of users [27]. Many existing tools for processing and analyzing remote sensing data require substantial technical knowledge, specialized software, or access to high-performance computing environments [28]. Consequently, non-expert users, including practitioners, decision-makers, and stakeholders in applied fields, often face challenges in obtaining timely and accurate information derived from vegetation indices or other spectral metrics [19,27]. Moreover, WebGIS platforms that integrate on-demand analytical capabilities remain limited, particularly those that allow dynamic computation of vegetation indicators directly from satellite data in an accessible online environment [29].

In light of these limitations, developing a WebGIS capable of performing automated vegetation index calculations presents an important and timely contribution [5]. By offering an intuitive interface, centralized data processing, and immediate delivery of analytical outputs, such a system can significantly broaden access to RS information and support a more inclusive decision-making process [19]. This approach not only reduces technical barriers but also enhances the practical utility of satellite-derived metrics across sectors such as environmental monitoring, agricultural management, and land-use planning [30]. Therefore, the creation of a user-centered WebGIS platform with integrated analytical functions is both justified and aligned with contemporary demands for accessible, scalable, and operational geospatial tools [18,19].

In line with the growing need to integrate more robust analytical measures into modern geospatial platforms, Rao’s Q Index emerges as a particularly relevant metric. This metric integrates information on richness, relative abundance, and the numerical magnitude and pairwise distances of pixel values within a multivariate analytical structure [31]. Conceptually, Rao’s Q represents the aggregate of all pairwise distances among pixel values in the examined satellite imagery, with each distance weighted by the relative abundance of the respective pixel pairs [32]. Such a formulation enables a more comprehensive and refined interpretation of the underlying data. In environmental research, Rao’s Q Index constitutes a systematic and robust approach for detecting temporal modifications in land-cover patterns [32,33,34]. The index is notably sensitive to subtle or small-scale variations and is particularly effective in capturing changes in soil and vegetation conditions by quantifying the degree of dissimilarity or similarity among samples obtained from distinct locations or time periods [5,14].

Unlike deep learning-based CD methods, which often require large volumes of labeled training data and substantial computational resources [35], the proposed approach leverages Rao’s Q Index to provide a statistically transparent and interpretable measure of spectral heterogeneity. This makes the platform particularly well suited for WebGIS applications, enabling efficient and reproducible change detection without the need for model training or specialized hardware.

The general objective of this study was to develop a WebGIS platform capable of rapidly detecting land cover changes across distinct insular regions, namely the Reykjanes Peninsula, through the implementation of Rao’s Q Index–based change-detection approaches. This system integrates and evaluates the performance of various spectral indices, providing an efficient and accessible environment for conducting post-eruptive spatial analyses.

## 2. Methods

### 2.1. Calculating Rao’s Q Index

Rocchini et al. [26] describe Rao’s Q as a quantitative descriptor designed to measure the degree of similarity or divergence among spatial configurations or distributions of ecological attributes. Spatial heterogeneity was quantified through the application of Rao’s Q Classic to the difference maps derived from pre- and post-eruption spectral data. The index was computed using a 9 × 9 pixel moving window, a parameterization that enabled the delineation of areas exhibiting pronounced spectral shifts attributable to fire disturbance [14,33,36]. The mathematical expression applied to derive Rao’s Q Index follows the formulation presented by Rocchini et al. [31,36] (Equation (1)).

(1) $$Rao’s\;Q=\sum d_{ij\;}\times p_{i}\times p_{j}$$

In this context, d*ij* denotes the spectral distance between pixel *i* and pixel *j*, whereas p*i* represents the proportion of pixel *i* in relation to the total pixels within an *n* × *n* window, expressed as p*i* = $\frac{1}{n^{2}}$.

The change detection (CD) using Rao’s Q is determined by the pixel values corresponding to the calculated change intensity [10], as described by Equation (2).

(2) $$Root\;Square\;Difference=\sqrt{{\left({Rao}_{After}-{Rao}_{Before}\right)}^{2}}$$

Expanding on the foundational contributions of Rocchini et al. [31,36,37,38], the present study extends the applicability of Rao’s Q beyond its established use in biodiversity and ecological monitoring by various spectral indices, within a freely accessible, open-source platform.

To extend the analytical framework beyond single-variable assessments, a multidimensional formulation of Rao’s Q was also employed. In this approach, multiple spectral indices were integrated simultaneously within the index’s computational structure. Prior to analysis, all indices underwent normalization to ensure dimensional comparability across variables. Pairwise dissimilarities were then estimated using a multidimensional distance metric applied within the same 9 × 9 pixel window. By incorporating complementary spectral information from several indices concurrently, this approach yielded a more comprehensive characterization of high-temperature-induced landscape alterations than would be achievable through any single index in isolation.

The support for two widely used satellite sensors, Sentinel-2 and Landsat 8/9, further broadens the operational scope of Rao’s Q approach, enabling its application to monitoring at a global scale across climatically and ecologically distinct environments, a context not previously explored in the literature.

### 2.2. Spectral Indices

Spectral indices are mathematical combinations of satellite reflectance bands designed to enhance specific surface features while suppressing background noise. Their formulation exploits the differential response of land cover types across the electromagnetic spectrum, enabling quantitative characterization of vegetation condition, water content, burned areas, and urban surfaces from remotely sensed imagery.

The developed application supports the computation of ten spectral indices (Table 1) organized across four thematic domains: vegetation condition, fire-affected area detection, water content, and urban land cover.

The vegetation domain comprises six indices: Normalized Difference Vegetation Index (NDVI) [39], Enhanced Vegetation Index (EVI) [40], Soil Adjusted Vegetation Index (SAVI) [41], Green Normalized Difference Vegetation Index (GNDVI) [42], and Normalized Difference Red Edge (NDRE) [43], each addressing specific limitations of the others, such as canopy saturation at high biomass, soil background interference, and reduced sensitivity to chlorophyll at advanced growth stages.

The fire detection domain includes the Normalized Burn Ratio (NBR) [44], Mid-Infrared Burn Index (MIRBI) [45], and Burned Area Index for Sentinel-2 (BAIS2) [46], which together cover a range of post-fire conditions from severe burn scars to low-intensity surface burns.

The Normalized Hotspot Indices (NHI) [47] was originally developed for volcanic thermal anomaly detection, and the algorithm has demonstrated consistent performance across a range of eruptive styles and intensities, from subtle degassing activity and low-flux lava flows to more vigorous effusive and explosive events, making it particularly well suited for the systematic monitoring of active volcanic systems [48].

Water content is assessed through the Normalized Difference Water Index (NDWI) [49], which captures liquid water absorption in the near-infrared region, while urban land cover is characterized by the Normalized Difference Built-up Index (NDBI) [50], which exploits the higher shortwave infrared reflectance of impervious surfaces relative to vegetated areas.

The indices selected represent methodological benchmarks within their respective domains, having been extensively validated across diverse geographic regions, climatic conditions, and land cover types in the peer-reviewed literature. Their inclusion reflects a deliberate design choice: rather than privileging a single analytical perspective, the application integrates complementary indices that collectively span the spectral sensitivity required for comprehensive environmental monitoring.

This multi-index framework ensures that the tool is not constrained to a narrow set of use cases but is instead capable of supporting a wide range of scientific investigations, from vegetation stress assessment and post-event recovery mapping to urban expansion analysis and surface water detection, within a single, coherent analytical workflow.

**Table 1:** Spectral indices implemented in the developed application, including their mathematical formulation, primary application domain, and original reference.

Spectral Index	Equation	Reference
NDVI (Normalized Difference Vegetation Index)	$\frac{NIR\;-\;Red}{NIR\;+\;Red}$	[51]
EVI (Enhanced Vegetation Index)	$G\times \frac{NIR\;-\;Red}{NIR\;+\;C1\;\times \;Red\;-\;C2\;\times \;Blue\;+\;L}$	[52]
NBR (Normalized Burn Ratio)	$\frac{\left(NIR\;-\;SWIR\right)}{\left(NIR\;+\;SWIR\right)}$	[53]
NDWI (Normalized Difference Water Index)	$\frac{Green\;-\;NIR}{Green\;+\;NIR}$	[49]
NDRE (Normalized Difference Red Edge)	$\frac{NIR\;-\;RedEdge}{NIR\;+\;RedEdge}$	[54]
SAVI (Soil Adjusted Vegetation Index)	$\frac{NIR\;-\;Red}{NIR\;+\;Red+L}\times (1+L)$	[41]
GNDVI (Green Normalized Difference Vegetation Index)	$\frac{NIR\;-\;Green}{NIR\;+\;Green}$	[42]
MIRBI (Mid-Infrared Burn Index)	10∗SWIR 2−9.8∗SWIR 1+2	[55]
BAIS2 (Burned Area Index for Sentinel-2)	$1-\left(\sqrt{\frac{B6\;*\;B7\;*\;B8A}{B4}}\right)*\left(\sqrt{\frac{B12\;-\;B8A}{B12\;+\;B8A}}\right)+1$	[46]
NDBI (Normalized Difference Built-up Index)	$\frac{SWIR\;-\;NIR}{SWIR\;+\;NIR}$	[50]
NHI (Normalized Hotspot Indices)	${NHI}_{1}=\frac{L_{2.2}\;-\;L_{1.6}}{L_{2.2}\;+\;L_{1.6}}$	[47]
	${NHI}_{2}=\frac{L_{2.2}\;-\;L_{0.8}}{L_{2.2}\;+\;L_{0.8}}$	

### 2.3. Threshold-Based Change Detection

The dissimilarity between each pair of Rao’s Q maps, both pre- and post-event, is represented by pixel values that quantify the estimated change intensity [36]. For each methodological approach (classic and multidimensional), a final binary map was generated, classifying areas into “change” and “no change” based on a determined threshold. This threshold was established using Otsu’s method [56], which determines an optimal cut-off by iterating over all possible threshold values and selecting the one that maximizes the inter-class variance between the “change” and “no change” populations in the difference map of pre- and post-event Rao’s Q Index values.

Otsu’s method was selected for the present analysis given its suitability for difference maps exhibiting bimodal distributions [57], a condition expected when comparing pre-event snow-covered surfaces with post-event lava-covered areas, where the two land cover states produce clearly distinct spectral responses in the SWIR domain [58,59].

The developed platform additionally offers the triangle method as an alternative thresholding approach [60], allowing users to adapt the binarization strategy to the spectral characteristics of different event types and land cover conditions [5,10,14].

### 2.4. Accuracy Assessment

The accuracy of the binary change maps generated by each Rao’s Q configuration was evaluated by computing the Overall Accuracy (OA), Producer’s Accuracy (PA), and User’s Accuracy (UA), as the proportion of correctly classified samples relative to the total number of assessed points. A stratified random sampling scheme was adopted, comprising 100 points generated in QGIS 3.10, equally distributed between the two classes: 50 points assigned to the “changed” category and 50 to the “no change” category. The validation was performed using the official lava flow data provided by the National Land Survey of Iceland [61], ensuring an independent reference for the accuracy assessment. Classification performance was interpreted according to the following agreement scale: below 40%—low agreement; 41–60%—moderate agreement; 61–75%—good agreement; 76–80%—excellent agreement; above 80%—almost perfect agreement [5,10,14,62].

### 2.5. Web-Based Platform

**Figure 1:**
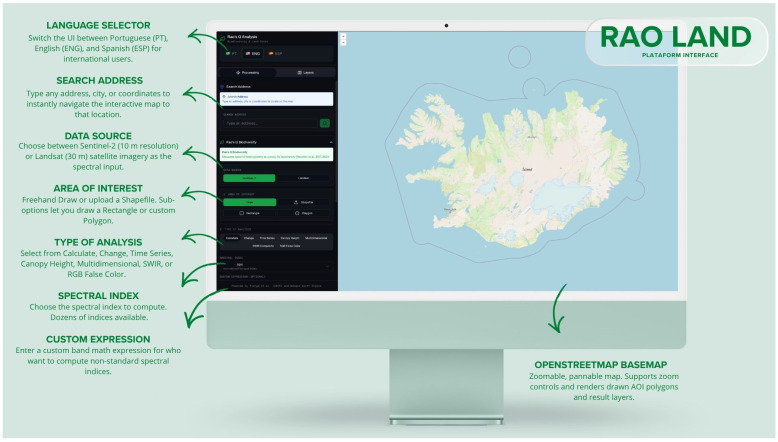
Overview of the RAO Land web platform interface, displaying the main control panel with options for data source selection (Sentinel-2 or Landsat 8/9), area of interest definition, type of analysis, and spectral index configuration, integrated with a Google Earth Engine backend and an OpenStreetMap basemap.

The proposed platform consists of an interactive WebGIS designed to support spatiotemporal analysis and CD using multispectral RS data. The system was developed with the aim of making advanced spectral diversity metrics, particularly Rao’s Q Index, accessible through an intuitive and user-friendly web interface (Figure 1).

The open-source application developed as part of this work was named “RAO Land” and is publicly available at https://www.rao-land.space/, where it may be accessed and used free of charge.

The web interface was implemented using the OpenLayers library, ensuring dynamic cartographic visualization, real-time interaction, and support for multiple geospatial layers. This approach enables efficient exploration of the results, including the visualization of thematic maps and comparison across different time periods.

Data processing is performed through an API that communicates with the GEE, leveraging its cloud-based computational capabilities and access to extensive multispectral image archives. Within this processing layer, the selected spectral indices are computed and the Rao’s Q-based algorithm is applied, enabling the quantification of spectral diversity and the identification of spatiotemporal change patterns.

This architecture, based on a clear separation between frontend and backend components, ensures scalability, computational efficiency, and reproducibility, making the platform suitable for both scientific research and operational environmental monitoring applications.

### 2.6. Backend Processing Pipeline

**Figure 2:**
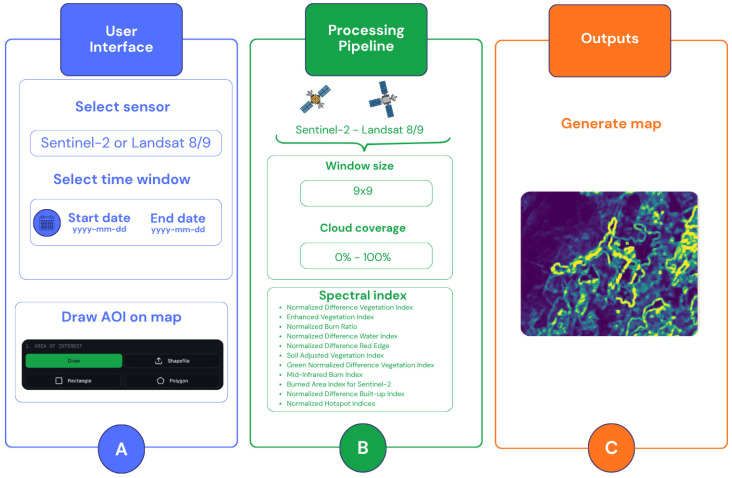
Schematic of the RAO Land application workflow. (**A**) User Interface (UI) with selection options. (**B**) Data processing pipeline including filter and spectral index selection to calculate Rao’s Q. (**C**) Outputs maps.

The backend processing pipeline constitutes the computational core of the developed application, responsible for retrieving satellite imagery, computing spectral heterogeneity measures, and delivering analytical outputs in a structured and reproducible manner (Figure 2).

Built upon the GEE cloud computing infrastructure, the pipeline leverages the processing capacity and data catalogue of one of the most widely adopted platforms in RS research, eliminating the need for local data storage or preprocessing and ensuring that analyses can be performed at any geographic location worldwide [63].

Image acquisition is constrained by a user-defined temporal window, specified through start and end dates, and by a cloud coverage threshold adjustable between 0% and 100%, allowing the user to control the quality and availability of the imagery according to the requirements of each analysis.

The spatial extent of the analysis is delimited by an AOI specified by the user through one of four supported input methods: freehand drawing, rectangular selection, polygon delineation, or shapefile upload.

Once the imagery is acquired, the pipeline proceeds to the computation of the selected spectral index over the defined AOI using a 9 × 9 pixel moving window, as recommended for Rao’s Q analyses of this nature [5,10,14,31,36,38,62]. The spectral distance among pixels within each window is calculated based on the reflectance values of the chosen index, and Rao’s Q is subsequently derived following the formulation described in the previous section. This process is applied independently to the pre- and post-event image composites, after which the change detection magnitude is computed as the root square difference between the two resulting Rao’s Q layers.

Upon completion of the processing, the pipeline delivers the outputs. These outputs consolidate satellite data acquisition, Rao’s Q plus spectral index computation, and result visualization within a single, integrated analytical workflow.

## 3. Use Case Demonstration

### 3.1. Study Area

**Figure 3:**
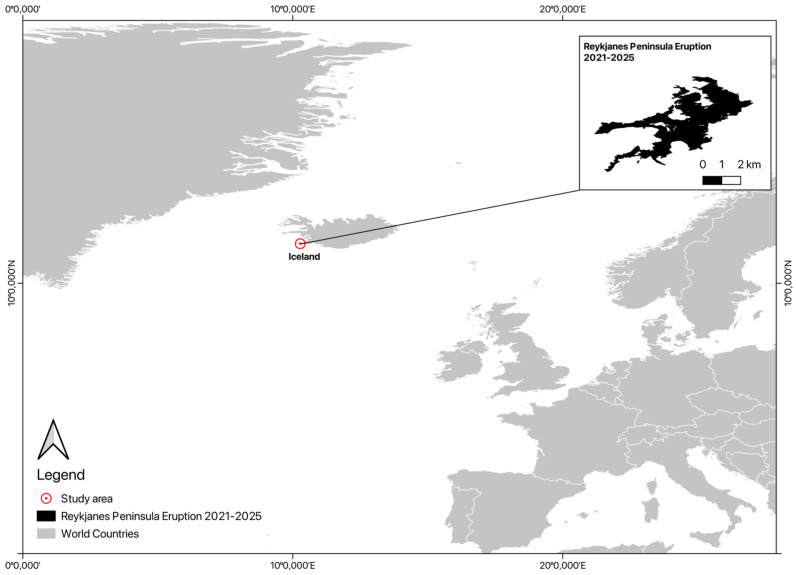
Study area location.

The study area is located in the Reykjanes Peninsula, southwestern Iceland, a volcanically active region situated on the Mid-Atlantic Ridge (Figure 3). This insular environment was selected due to its geophysical significance and the occurrence of an eruptive episode in February 2024, following a period of volcanic unrest that began in late 2023.

Two multispectral images acquired by the Sentinel-2 satellite were used: one from February 2022, serving as the baseline reference period, and one from February 2024, corresponding to the active eruptive phase. Both images were acquired under comparable seasonal conditions, minimizing phenological and illumination biases in the spectral analysis.

**Figure 4:**
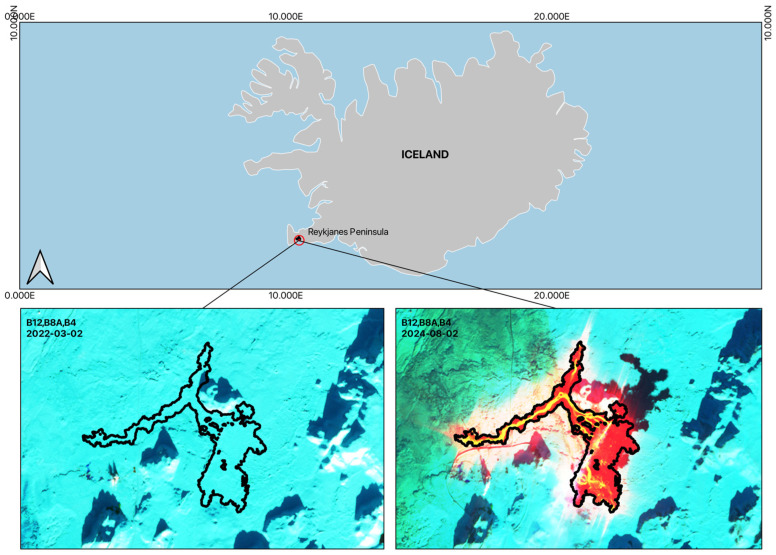
False-color composite images illustrating land surface changes associated with the volcanic eruption. Pre-eruptive conditions (left) show a landscape dominated by snow, ice, and sparse rocky outcrops. Post-eruptive conditions (right) are marked by the emergence of active lava flows with high thermal radiance, solidified lava fields, degassing plumes, and newly exposed volcanic substrate, reflecting the substantial transformation of the land surface driven by eruptive activity.

The false-color composites (Figure 4) clearly illustrate the contrast between the pre-eruptive landscape, dominated by snow and ice cover with sparse rocky outcrops, and the eruptive scenario, characterized by active lava flows displaying high thermal radiance, solidified lava fields, degassing plumes, and exposed volcanic substrate.

The SWIR false-color composite (Figure 4) enables clear delineation of the lava flow emplaced during the eruptive episode of 8–9 February 2024, as the shortwave infrared bands effectively highlight thermal anomalies and freshly solidified volcanic material against the surrounding snow and ice-covered landscape.

The spatial extent of the lava flow derived in this study shows strong agreement with the official lava flow boundary mapped by the National Land Survey of Iceland [61] on 13 February 2024, four days after the eruptive event. This correspondence between the remotely sensed results and the independently produced cartographic boundary serves as a validation of the methodology applied in this work, demonstrating the capability of SWIR-based analysis to accurately capture lava flow extent in near-real-time volcanic monitoring scenarios.

### 3.2. Results

The present study applies the Rao’s Q method to Sentinel-2 MSI sensor imagery, using SWIR bands: band 11 (1610 nm) and band 12 (2190 nm), as well as a multidimensional combined index (band 11 + band 12) and the NHI proposed by Genzano et al. [47]. These SWIR bands were selected due to their high sensitivity to thermal emissions, making them particularly effective for detecting areas affected by volcanic activity [64].

Two images were used: a reference image acquired on 3 February 2022 (no eruption, snow-covered ground) and an analysis image acquired on 8 March 2024 (active eruption, ground equally snow-covered outside the lava flow zone).

The analysis was conducted under four configurations:

(i) classic Rao’s Q using band 11 (SWIR 1);
(ii) classic Rao’s Q using band 12 (SWIR 2);
(iii) multidimensional Rao’s Q combining bands 11 and 12;
(iv) classic Rao’s Q using the NHI defined as NHI = (B12 − B11)/(B11 + B12).

Result validation was carried out using the official lava flow area polygons provided by the National Land Survey of Iceland [61], employed as ground truth reference data. Accuracy was assessed through a confusion matrix comprising 100 samples (Table 2), from which OA, PA, and UA were computed.

#### 3.2.1. Rao’s Q Using 1.6 µm (SWIR 1) Wavelength

**Figure 5:**
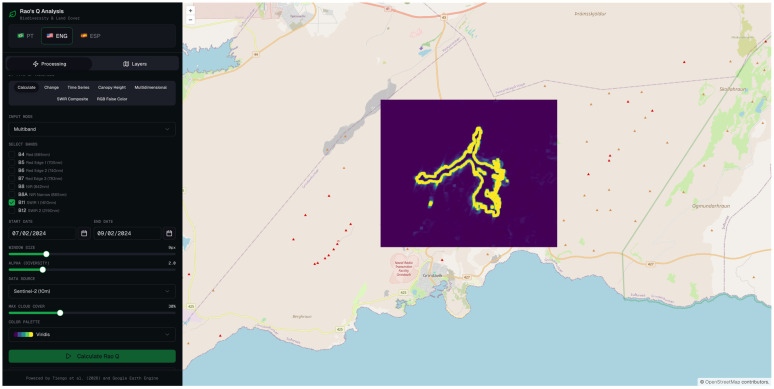
Spatial output of the Rao’s Q change detection analysis applied to Sentinel-2 MSI band 11 (SWIR 1).

The spatial distribution of the detected changes is illustrated in Figure 5, which presents the Rao’s Q output for band 11 (SWIR 1) over the study area. The result reveals a well-defined thermal anomaly spatially coincident with the lava flow field emplaced during the March 2024 eruptive episode, thereby confirming the capability of the method to delineate active volcanic surfaces under snow-covered conditions.

**Figure 6:**
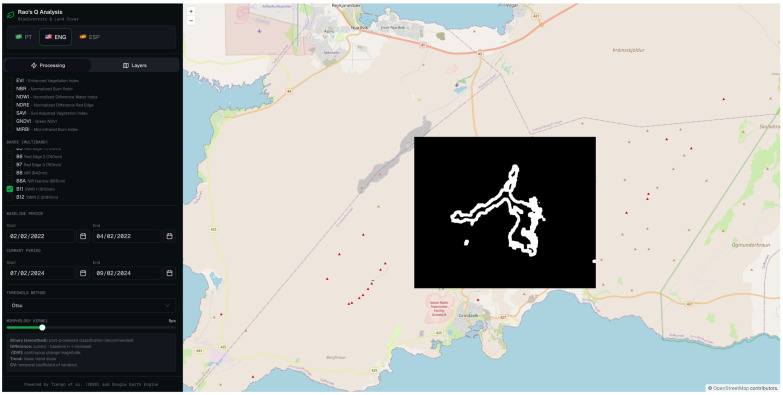
Binary classification output of the Rao’s Q change detection analysis applied to Sentinel-2 MSI band 11 (SWIR 1).

The binary classification map derived from the Rao’s Q analysis is presented in Figure 6, which spatially delineates the pixels classified as changed (white) and unchanged (black) between the reference and analysis acquisition dates. The resulting classification reveals a coherent and spatially continuous changed area whose geometry and extent are consistent with the known lava flow field emplaced during the March 2024 eruptive episode. The delineated surface exhibits the characteristic elongated morphology of channeled lava flows, with a well-defined primary axis and secondary lobes extending towards the flow margins.

The Rao’s Q method applied to band 11 yielded the best overall results, with an OA of 86.0%, a UA of 97.4%, and a PA of 74.0% (Table 2). The exceptionally high UA indicates that nearly all pixels classified as “changed” genuinely correspond to the lava flow, reflecting an extremely low false positive rate. This behavior is consistent with the underlying physics: band 11 is highly sensitive to thermal emission from heated surfaces (such as fresh or recently cooled lava), whereas snow exhibits very low reflectance at this wavelength, thereby maximizing the spectral contrast between the two acquisition dates [65].

The PA of 74.0%, while not the highest within the set, indicates that approximately 26% of actual changes went undetected (false negatives), which may be attributed to areas of the lava flow where surface temperature had already decreased by the time of image acquisition, or where lava thickness was insufficient to produce a significant alteration in the SWIR 1 signal.

#### 3.2.2. Rao’s Q Using 2.2 µm (SWIR 2) Wavelength

**Figure 7:**
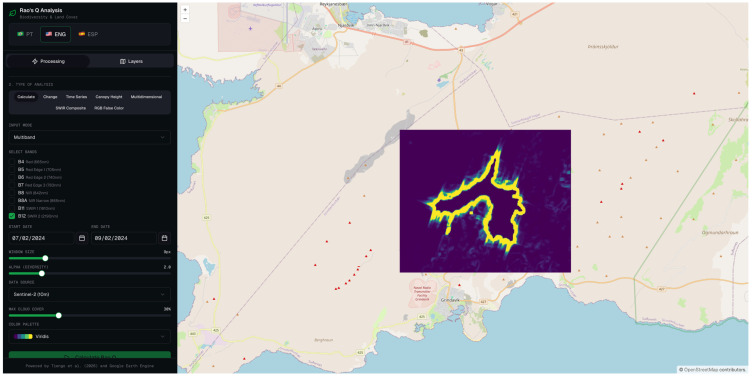
Continuous change magnitude map produced by Rao’s Q method using Sentinel-2 MSI band 12 (SWIR 2).

As shown in Figure 7, the continuous change magnitude map reveals an elevated thermal signal distributed across the lava flow field, with maximum intensities concentrated along the primary flow channel. However, a notable characteristic of this configuration is the wider spatial extent of intermediate-magnitude change values along the flow periphery, suggesting that the SWIR 2 band captures a broader thermal halo around the active flow, potentially including areas of residual ground heating or thermally disturbed snow at the lava-snowpack interface.

**Figure 8:**
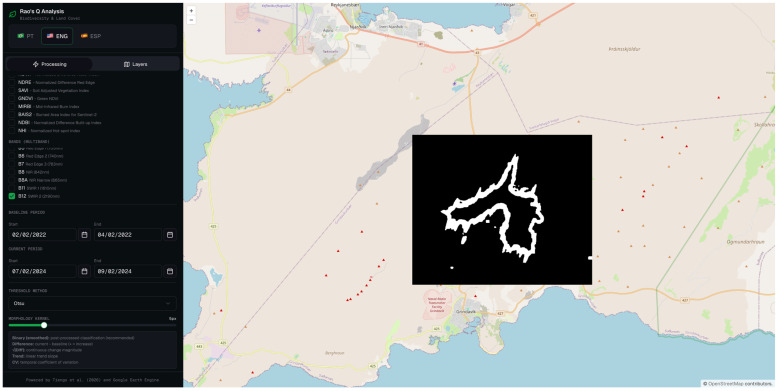
Binary change map derived from the Rao’s Q analysis of Sentinel-2 MSI band 12 (SWIR 2) for the same acquisition pair. White pixels denote areas classified as changed, and black pixels denote unchanged areas.

The binary classification map (Figure 8) translates this signal into a discrete delineation of the affected area. Unlike the spatially coherent and morphologically consistent output obtained for band 11, the band 12 binary map exhibits a markedly fragmented and irregular classification pattern. This spatial incoherence suggests that the SWIR 2 signal was insufficiently discriminant between the thermal anomaly of the lava flow and the background spectral variability introduced by the surrounding snow-covered terrain, leading to a classification that is neither spatially compact nor geometrically consistent with the known emplacement of the March 2024 lava flow.

The performance of Rao’s Q using band 12 was substantially inferior, with an OA of only 46.0%, a PA of 16.0%, and a UA of 40.0% (Table 2). These values, at or below the random chance threshold, suggest that band 12 was unable to effectively discriminate the lava flow from the snow-covered background under the specific conditions of this image pair. The primary explanatory factor lies in the greater sensitivity of band 12 to soil moisture and liquid water content [66] within the snowpack, which may have introduced inter-date spectral variations unrelated to volcanic activity, thereby generating a high rate of both false positives and false negatives.

#### 3.2.3. Rao’s Q Using NHI

**Figure 9:**
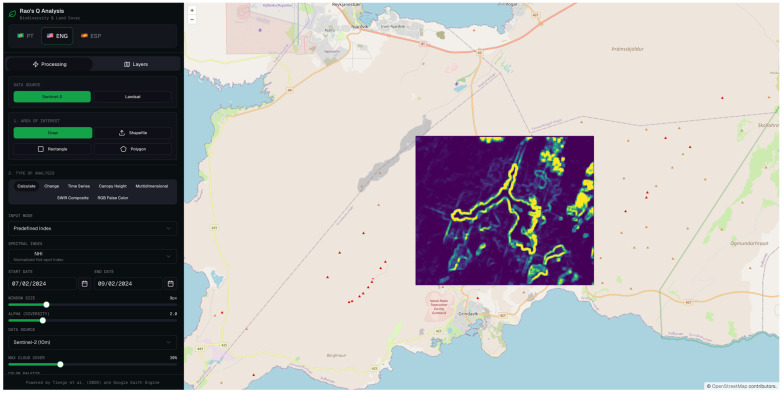
Rao’s Q method applied to the Normalized Heat Index (NHI) derived from Sentinel-2 MSI imagery.

Figure 9 presents the classical Rao’s Q method is applied to the NHI. The resulting map reveals an elevated change signal broadly coincident with the lava flow field, yet with a notably more diffuse spatial pattern compared with the band 11 output, with intermediate-magnitude values extending considerably beyond the primary flow boundaries into the surrounding terrain.

**Figure 10:**
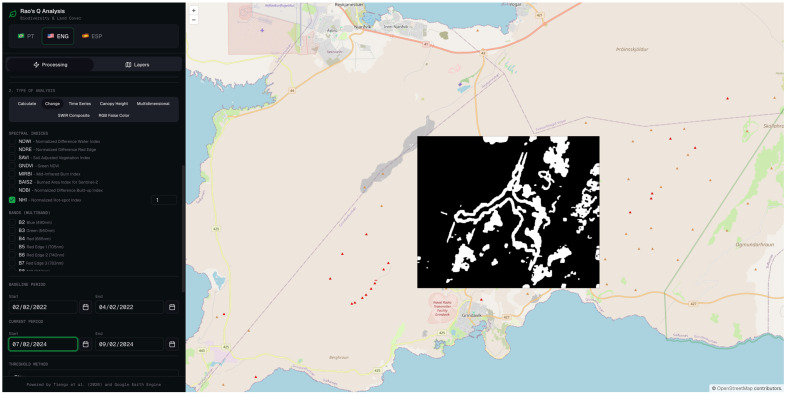
Binary change map derived from the classical Rao’s Q method applied to the Normalized Heat Index (NHI). White pixels denote areas classified as changed, and black pixels denote unchanged areas.

However, visual inspection of the NHI change detection map (Figure 10) reveals a higher density of false positives outside the official lava flow boundaries compared with the band 11 configuration. This tendency is consistent with the greater susceptibility of the NHI to variations in surface moisture and snow phenological changes, which preferentially affect band 12 [47].

The NHI configuration achieved an OA of 80.0%, a PA of 72.0%, and a UA of 85.7% (Table 2). The high UA confirms a low incidence of false positives, while the PA of 72.0% indicates that approximately 28% of actual lava flow pixels went undetected, likely associated with areas of weaker thermal emission at the flow margins or zones of thinner lava deposition. Although these results are competitive, they fall short of the band 11 configuration across all metrics, suggesting that the normalization introduced by the NHI partially attenuates the thermal signal that Rao’s Q relies upon for change detection.

#### 3.2.4. Rao’s Q MD Using 1.6 µm (SWIR 1) and 2.2 µm (SWIR 2) Wavelength

**Figure 11:**
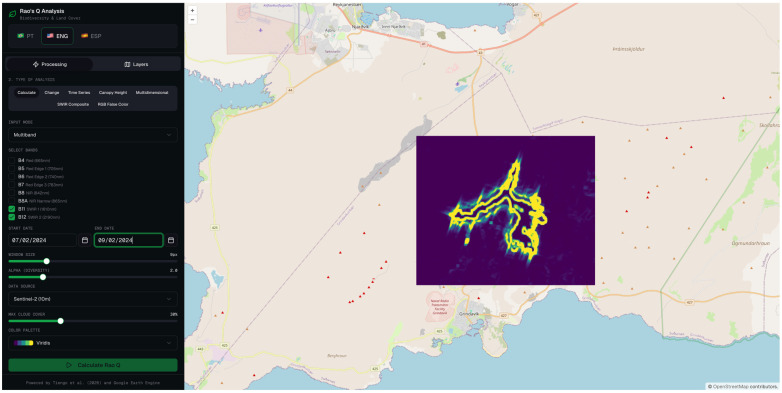
Rao’s Q multidimensional method combining Sentinel-2 MSI bands 11 (SWIR 1) and band 12 (SWIR 2).

The multidimensional Rao’s Q configuration simultaneously exploits the spectral information of bands 11 and 12, jointly capturing the complementary sensitivity profiles of the two SWIR bands to thermally anomalous surfaces. The resulting change magnitude map (Figure 11) reveals an elevated thermal signal distributed across the lava flow field, with maximum change intensities concentrated along the primary flow channel and progressively diminishing towards the lateral margins and flow front.

**Figure 12:**
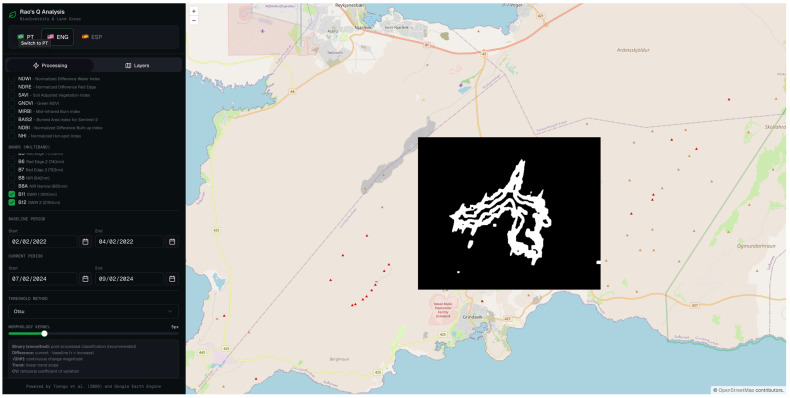
Binary change map derived from the multidimensional Rao’s Q method combining bands 11 and 12, for the same acquisition pair. White pixels denote areas classified as changed, and black pixels denote unchanged areas.

The binary classification map (Figure 12) translates this continuous signal into a discrete spatial delineation of the affected area. The classified region successfully captures the primary lava flow body as well as a number of secondary flow structures, producing a morphologically articulated output whose overall geometry is broadly consistent with the expected extent of the lava flow field.

This multidimensional approach, combining bands 11 and 12, produced a remarkably balanced set of metrics: OA of 83.0%, PA of 84.0%, and UA of 82.4% (Table 2). This is the only method that presents a balance between sensitivity and specificity without pronounced sacrifice of any of the metrics, which is particularly relevant in emergency applications where both the omission and commission of changes have operational consequences.

## 4. Discussion

The findings of this study demonstrate that the developed WebGIS platform constitutes a viable and effective solution for the rapid detection and spatial characterization of land-cover changes in volcanically active insular environments. By leveraging cloud-based computational infrastructures, namely GEE, the system was capable of processing extensive satellite imagery datasets with notable efficiency, substantially reducing the time typically required by conventional desktop-based workflows [67,68,69]. This operational speed is particularly relevant in post-eruptive monitoring scenarios, where the timely availability of geospatial outputs can directly inform emergency response and territorial management decisions.

Beyond computational performance, the platform was designed with accessibility as a central principle. By offering an intuitive web-based interface that requires no specialized desktop software or advanced programming proficiency, the system addresses one of the most persistent barriers to the operational adoption of RS technologies among non-expert users. Practitioners, land managers, and decision-makers with limited technical backgrounds can interact with the analytical environment and obtain meaningful spectral outputs without engaging directly with the underlying data processing routines. This approach aligns with an increasingly recognized need in the geospatial community to develop tools that democratize access to satellite-derived information and extend its utility beyond the academic and technical sectors.

The architecture of the platform incorporates a diverse set of spectral indices, each calibrated for distinct analytical contexts and land-cover conditions. This modular design reflects the recognition that no single spectral metric is universally optimal across all environmental scenarios. Indices sensitive to vegetation canopy dynamics, surface water extent, urban built-up density, and soil reflectance characteristics are made available within the same operational environment, enabling users to select the most appropriate analytical tool according to the specific nature of the phenomenon under investigation. Such flexibility enhances the broader applicability of the system and positions it as a multipurpose geospatial instrument capable of supporting a wide range of monitoring and assessment tasks.

The joint analysis of the four configurations enables the establishment of a performance hierarchy conditioned by the intended application objective. Where the goal is to minimize false positives (high precision), the classical Rao’s Q applied to band 11 is clearly superior, achieving a UA of 97.4%. Where the goal is to maximize the detection of all actual changes (high sensitivity/recall), the multidimensional Rao’s Q combining band 11 and band 12 is preferable, with a PA of 84.0%. The NHI offers an intermediate trade-off, with the drawback of greater spatial noise, while band 12 in isolation proved inadequate for this specific scenario.

The snow cover conditions present in both images were a determining factor in the results obtained. Snow acts as a spectrally neutral background in the SWIR region, exhibiting very low reflectance, which maximizes the contrast introduced by the lava and minimizes the influence of seasonal vegetation changes, one of the primary confounding factors in change detection [66]. In this respect, the results obtained here represent a favorable scenario, and it is to be expected that method performance will be lower in contexts characterized by heterogeneous vegetation cover or adverse atmospheric conditions.

The SWIR RGB composite of the 8 March 2024 image visually confirms the presence of active and recently emplaced lava within the area delineated by the official polygons, with high-temperature cores (white/bright) and zones of cooled lava (dark red), consistent with the detection patterns obtained by the classical Rao’s Q band 11 and the multidimensional configuration. The existence of lava areas outside the official boundary, visible as residual red patches in the SWIR composite, may account for a portion of the false negatives recorded in configurations with lower PA values.

The reliance on GEE as the primary data processing infrastructure proved to be a foundational element of the platform’s performance. The capacity to access and process large-scale satellite archives without requiring local data storage or high-performance computational hardware substantially lowered the infrastructural barriers associated with remote sensing analysis [1,63]. The integration of cloud-based processing into a WebGIS framework thus not only enhances analytical efficiency but also contributes to the broader goal of making advanced geospatial methodologies more equitably accessible across different institutional and geographic settings [70,71,72].

Although the analytical focus of this study was directed at the Reykjanes Peninsula, the platform’s modular architecture and the diversity of available spectral indices confer upon it a degree of transferability to other insular or continental environments subject to distinct forms of land-cover disturbance. The selection of the Reykjanes Peninsula as the study area provided a demanding and analytically informative test case, given the rapidity, spatial extent, and spectral complexity of the surface changes induced by recent volcanic eruptions. The capacity of the system to produce reliable and spatially coherent outputs under such conditions suggests that its applicability may extend to other high-disturbance environments, including areas affected by wildfire [5], flooding [73], coastal erosion [74], or multihazards [75], provided that appropriate spectral metrics are selected for each specific context.

In sum, the results of this study affirm the analytical and operational value of integrating cloud-based RS processing with an accessible WebGIS environment for post-eruptive change detection. The combination of a flexible multi-index framework, rapid processing capabilities, and an inclusive UI positions the developed platform as a meaningful contribution to the expanding field of operational geospatial monitoring. The effective application of both the NHI and Rao’s Q Index in the Reykjanes Peninsula context illustrates how the deliberate alignment of spectral metrics with the physical properties of the phenomenon under study can yield analytically coherent and practically relevant outputs. These findings lay a foundation for future development oriented toward enhanced validation, expanded index integration, and broader deployment across diverse environmental monitoring scenarios.

## 5. Conclusions

This study presented the development and evaluation of a cloud-based WebGIS platform for the detection and spatial characterization of LULCC in volcanically active environments, with the Reykjanes Peninsula, Iceland, serving as the primary test case. The integration of GEE as the core computational infrastructure enabled the efficient processing of large-scale satellite imagery archives, substantially reducing the time and technical overhead associated with conventional desktop-based workflows. The resulting platform demonstrated operational viability as a rapid-response geospatial tool applicable to post-eruptive monitoring scenarios.

The accessibility-oriented design of the platform, grounded in an intuitive web-based interface that requires no specialized software or programming expertise, represents a meaningful contribution toward the democratization of RS technologies. By enabling non-expert users to obtain analytically relevant spectral outputs without direct engagement with underlying data processing routines, the system addresses a persistent barrier to the broader operational adoption of satellite-derived geospatial information.

The platform’s modular architecture and diverse spectral index framework confer a degree of transferability beyond the volcanic context, suggesting potential applicability to other high-disturbance environments, including areas affected by wildfire, flooding, coastal erosion, or multihazard events, provided that spectrally appropriate metrics are selected for each specific context.

The results demonstrate that the Rao’s Q method is effective in detecting lava flows from Sentinel-2 SWIR imagery, with performance dependent on the spectral band employed. The Rao’s Q applied to band 11 distinguished itself through high precision (UA = 97.4%) and is recommended in contexts where the spatial reliability of the classification output is critical. The multidimensional approach (band 11 + band 12) proved to be the most balanced configuration (OA = 83.0%, PA = 84.0%, UA = 82.4%) and is preferable for operational emergency mapping purposes. Band 12 in isolation proved inadequate for this context, while the NHI of Genzano et al. [47] yielded acceptable performance but with greater spatial noise.

The combined use of bands 11 and 12 yielded the most balanced performance among the tested spectral metrics, with all accuracy indicators (OA, PA, and UA) exceeding 80%. This consistency highlights the robustness of the multidimensional SWIR combination for detecting lava-affected areas.

It should be noted that the results presented here derive from a single case study conducted under relatively favorable and spectrally homogeneous conditions. Therefore, the applicability of the proposed approach to other environmental change contexts, such as forest fires, floods, or coastal erosion, should be regarded as a promising research direction rather than a demonstrated outcome. Future work involving diverse case studies and more spectrally heterogeneous environments will be essential to confirm the generalizability of the methodology.

Future developments of the platform will prioritize the integration of Sentinel-1 SAR data, given its immediate relevance for scenarios in which optical sensors are compromised by cloud cover or volcanic smoke. Nevertheless, it is important to note that, during the course of an eruption, SAR data do not provide direct information on the location of thermally active areas, such as active lava flows or eruptive vents. Its contribution is therefore largely complementary, serving primarily as an indicator of land cover changes rather than as a tool for identifying ongoing volcanic activity. For this reason, the combined use of SAR and optical data is envisioned as the most effective strategy for ensuring continuous monitoring under adverse atmospheric conditions.

In conclusion, the findings of this study affirm the analytical and operational value of integrating cloud-based RS processing with an accessible WebGIS environment for post-eruptive land-cover monitoring. Future work should be directed toward enhanced accuracy assessment through independent validation datasets, expanded integration of additional spectral indices and sensor sources, and broader deployment across diverse environmental monitoring scenarios, with the aim of consolidating the platform as a robust and widely applicable geospatial monitoring instrument.

## Figures and Tables

**Table 2:** Accuracy results for each Rao’s Q methodological approach.

Spectral Metrics	OA (%)	PA (%)	UA (%)
Band 11	86.0	74.0	97.4
Band 12	46.0	16.0	40.0
NHI	80.0	72.0	85.7
Band 11 + Band 12	83.0	84.0	82.4

## Data Availability

The original contributions presented in this study are included in the article. Further inquiries can be directed to the corresponding author.
